# Supplementary material for: Intrafusal-fiber LRP4 for muscle spindle formation and maintenance in adult and aged animals
Source: Nat Commun. 2023 Feb 10;14:744. doi: 10.1038/s41467-023-36454-8 (PMC9918736; doi:10.1038/s41467-023-36454-8)
Supplement: Supplementary file 1 — Supplementary Information [file 41467_2023_36454_MOESM1_ESM.pdf]

Supplementary Information

Intrafusal-fiber LRP4 for muscle spindle formation and maintenance in adult and aged animals

Rangjuan Cao[1,2#], Peng Chen[1#], Hongsheng Wang[1#], Hongyang Jing[1], Hongsheng Zhang[1], Guanglin Xing[1], Bin Luo[1], Jinxiu Pan[1], Zheng Yu[1], Wen-Cheng Xiong[1,3*], Lin Mei[1,3*]

[1]Department of Neurosciences, School of Medicine, Case Western Reserve University, Cleveland, OH 44106, USA

[2]Department of Hand and Foot Surgery, China-Japan Union Hospital of Jilin University, Changchun, China

[3]Louis Stokes Cleveland Veterans Affairs Medical Center, Cleveland, OH 44106, USA

Running title: LRP4 for muscle spindle formation and maintenance

[#] These authors contributed equally

***Correspondence**

Lin Mei, lin.mei@case.edu

Wen-Cheng Xiong, wxx119@case.edu

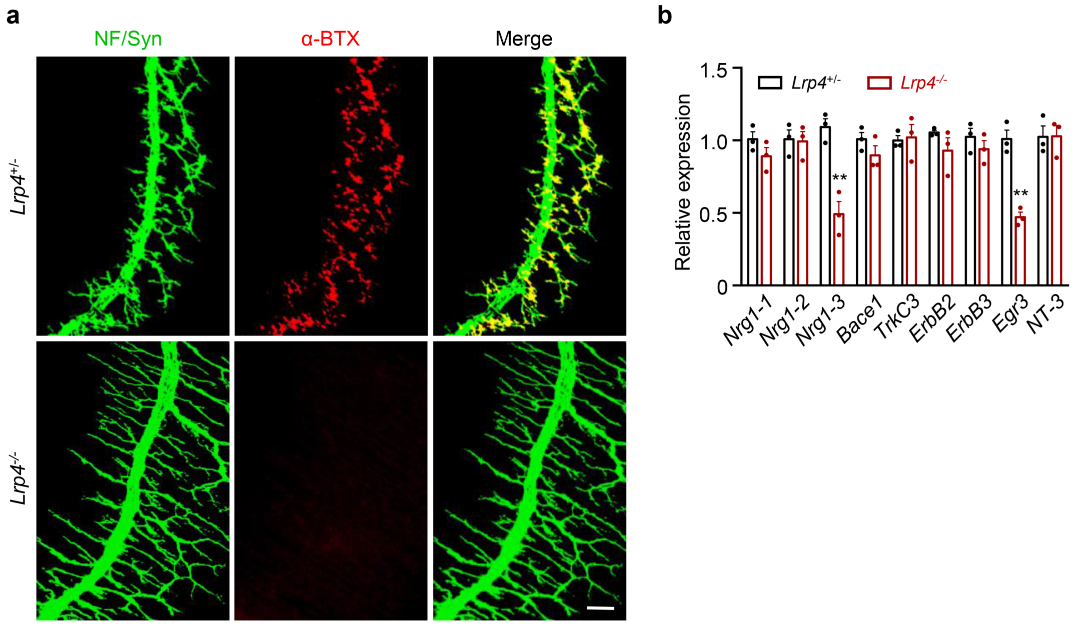

Supplementary Fig.1 Impaired NMJs and altered gene expression in *Lrp4* mutant. a Images of left ventral diaphragms of *Lrp4*$^{+/-}$ or *Lrp4*$^{-/-}$ labeled with NF/Syn (green) and α-BTX (red) at E14.5. Axon overshooting with no regular NMJs in *Lrp4*$^{-/-}$. Scale bar, 100 μm. **b** Quantitative RT-PCR analyzed the relative expression of *Nrg1-1*, *Nrg1-2*, *Nrg1-3*, *Bace1* and *TrkC* in DRG, as well as *ErbB2*, *ErbB3*, *Egr3* and *NT-3* in muscle in indicated group at E18.5. Data are shown as mean ± SEM. n = 3 mice per group, unpaired two-tailed *t* test, $^{**}p = 0.0043$ for *Nrg1-3*, $^{**}p = 0.0016$ for *Egr3*. Source data are provided as a Source Data file.

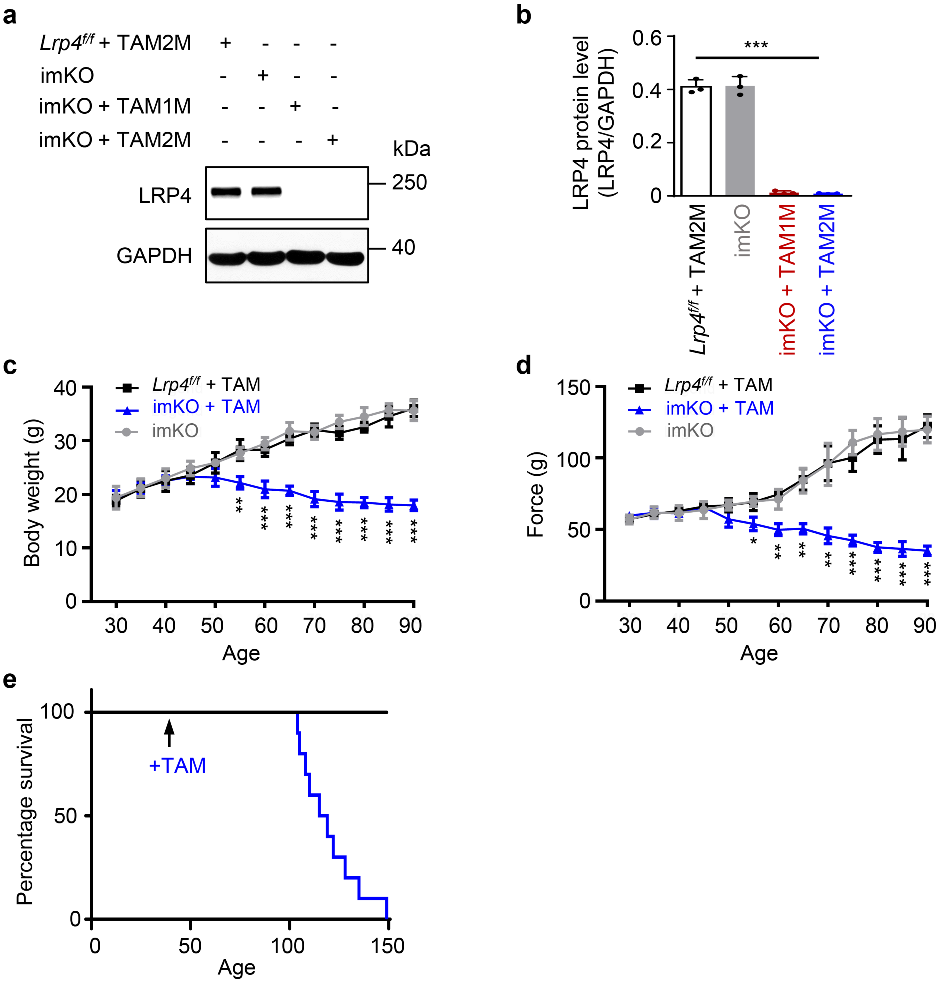

Supplementary Fig. 2 Reduced muscle strength and body weight in conditional *Lrp4* knockout adult. a Reduced expression of LRP4 in tamoxifen treated mice. Western blotting tested the expression of LRP4 in indicated group. Uncropped blots in Source Data. **b** Quantification of the relative expression of LRP4 protein related to GAPDH; F (3, 8) = 325.4, ***p < 0.0001. n = 3 per group, one-way ANOVA. **c** Body weight loss after tamoxifen treatment. The curve of body weight with mice age in tamoxifen treated imKO mice, tamoxifen treated *Lrp4*$^{f/f}$ or no tamoxifen treated imKO mice; **p = 0.0037 for P55, ***p = 0.0004 for P60, ***p < 0.0001 for P65 to P90. n = 3 mice per genotype, one-way ANOVA. **d** Decreased grip strength in tamoxifen treated imKO mice. The curve of grip strength with mice age in each group; *p = 0.0125 for P55, **p = 0.0019 for P60, **p = 0.0013 for P65, **p = 0.0024 for P70, ***p < 0.0001 for P75 to P90. n = 3 mice per genotype, one-way ANOVA. **e** Kaplan-Meier survival curves of control and tamoxifen treated imKO mice. χ^2 = 10, **p = 0.002. n = 10 mice per group, Log-Rank (Mantel-Cox) test. Data are shown as mean ± SEM. Source data are provided as a Source Data file.

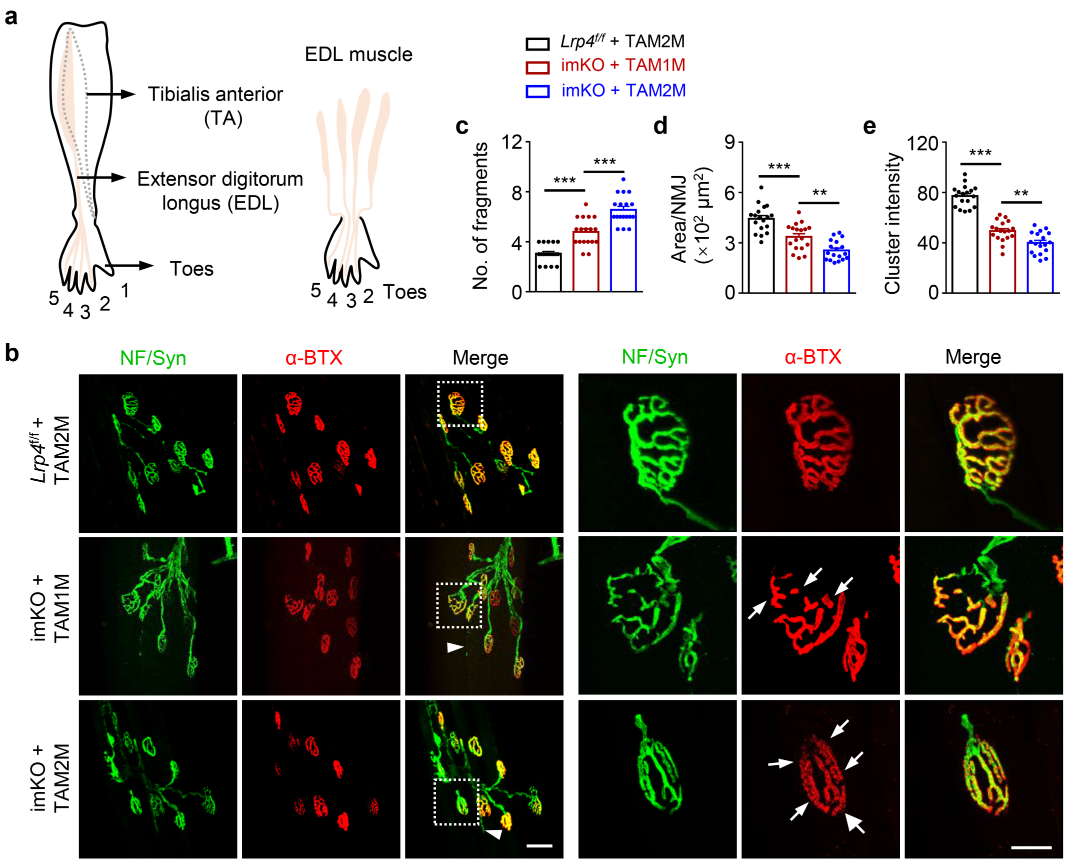

Supplementary Fig. 3 Conditional knockout of *Lrp4* in adult leads to NMJs deficient. **a** Schematic diagram of EDL muscle. EDL muscle consists of four smaller pieces, each of which attaches a specific toe. **b** Represent images of EDL muscles of indicated genotypes (P60, P90), whole mount stained with NF/Syn (green) and α-BTX (red) to label axon terminals and AChRs. Arrows, fragmentation of NMJs in tamoxifen treated imKO mice, arrowheads; axon overshooting; square, NMJs enlarged in the right panel. Scale bars, 50 μm and 20 μm. **c-e** Quantitative analysis of data in (**b**). **c** More fragmented AChR clusters in tamoxifen treated imKO mice; $t_{(34)} = 5.495$, ***$p < 0.0001$; $t_{(34)} = 4.712$, ***$p < 0.0001$.**d** Reduced area per NMJs in imKO mice treated by tamoxifen; $t_{(34)} = 4.041$, ***$p = 0.0003$; $t_{(34)} = 3.507$, **$p = 0.0013$. **e** Decreased AChR intensity (measured by mean pixel value) in *Lrp4* conditional knockout mice; $t_{(34)} = 10.06$, ***$p < 0.0001$; $t_{(34)} = 3.35$, **$p = 0.002$. n = 18 NMJs from 3 mice per group, unpaired two-tailed t test in (**c-e**). Data are shown as mean ± SEM. Source data are provided as a Source Data file.

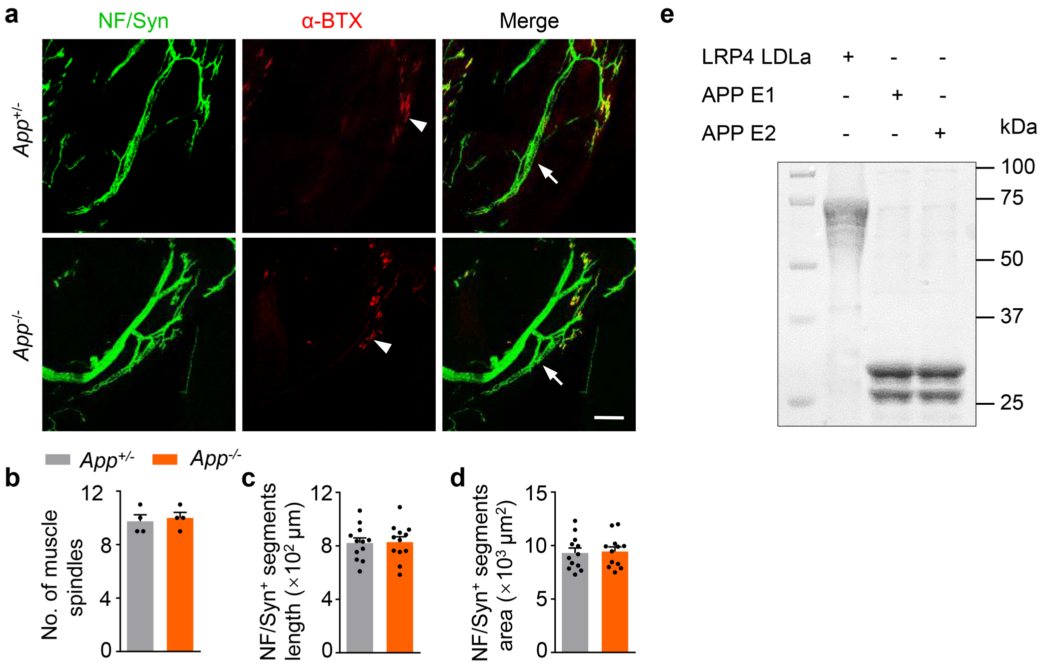

Supplementary Fig. 4 Normal muscle spindle in *App null* mice. a No morphological deficiency of Muscle spindle in *App*$^{-/-}$. Representative images of muscle spindle in *App*$^{+/-}$ and *App*$^{-/-}$ mice at E18.5 stained with NF/Syn (green) and α-BTX (red). Arrowheads, regular NMJs; Arrows, muscle spindles. Scale bar, 100 µm. **b-d** Quantification of data in (**a**). No difference in number of muscle spindle (**b**), NF/Syn^{+} segments length (**c**), or area of NF/Syn^{+} nerve covering (**d**) between *App*$^{+/-}$ and *App*$^{-/-}$. Data shown as mean ± SEM, n = 4 mice (**b**) or 12 muscle spindles from 4 mice (**c, d**) per group, unpaired two-tailed *t* test. **e** Coomassie blue staining of the purified LRP4 LDLa, APP E1 and APP E2 recombinant proteins. 10 µL of recombinant proteins were separated by SDS-PAGE and stained with Coomassie blue. Source data are provided as a Source Data file.

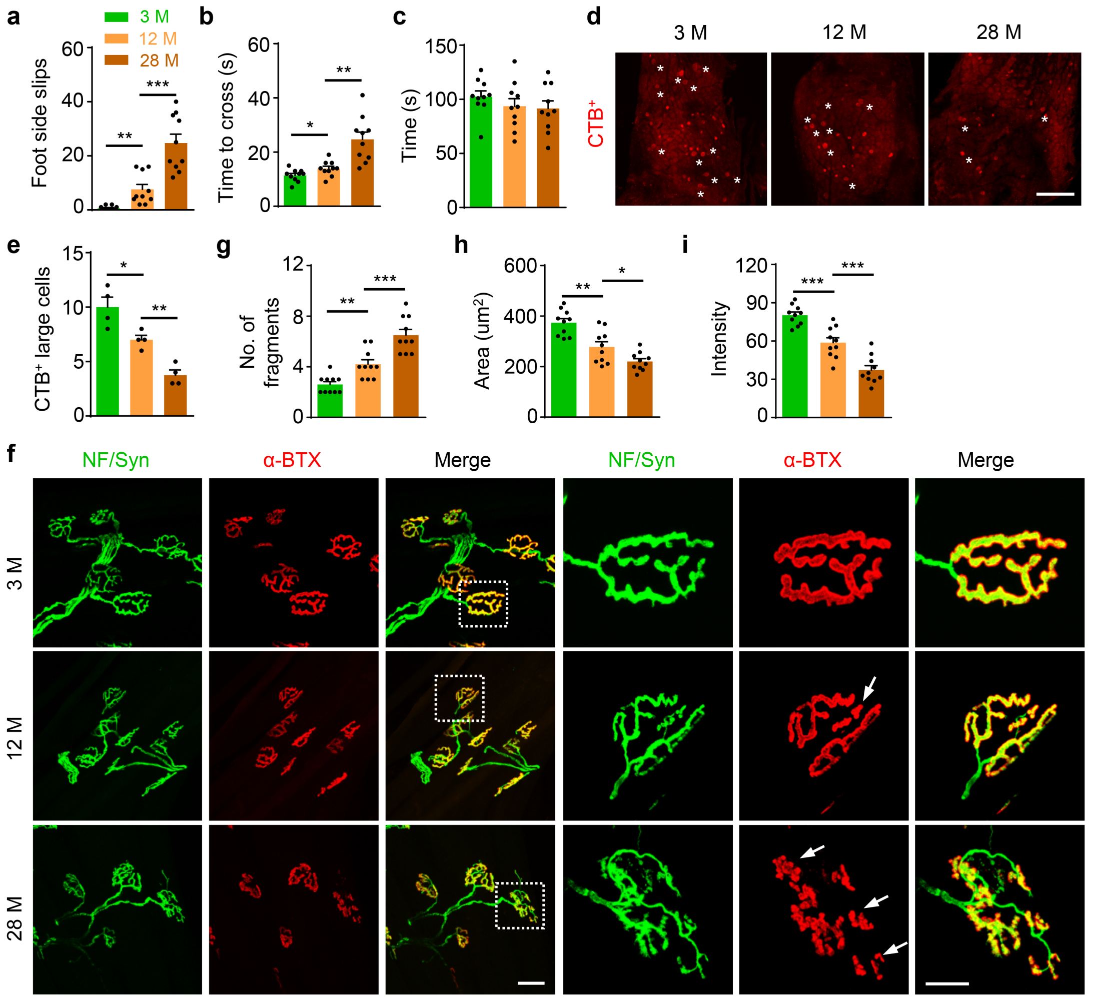

Supplementary Fig. 5 Compromised coordinative movement and fragmented NMJs in the aged mice. a Quantification of number of side slips in beam walking task on beam of 6 mm in each group. Increased side slips in 12 M compared with 3 M; $t_{(18)} = 3.804$, **$p = 0.0013$. More in 28 M compared with 12 M; $t_{(18)} = 4.541$, ***$p = 0.0003$. **b** Quantification of time required in beam walking task. More time was required for 12 M compared with 3 M mice; $t_{(18)} = 2.209$, *$p = 0.04$, and longer time in 28 M compared with 12 M; $t_{(18)} = 3.87$, **$p = 0.001$. **c** Quantification of the time mice on the rotating rod at 3M, 12M, and 28M-old age. n = 10 mice per group, unpaired two-tailed *t* test in (**a-c**). **d** Representative images of DRG whole mount stained with anti-CTB antibody (red) after CTB virus injection. Asterisks, large CTB-positive neurons, scale bar, 300 μm. **e** Quantification of CTB labeled neurons with soma size larger than 1000 μm^2. $t_{(6)} = 3$, *$p = 0.024$; $t_{(6)} = 5.166$, **$p = 0.002$. n = 4 mice per group, unpaired two-tailed *t* test. **f** Increased fragmentation and denervation in the aged mice. Representative images of EDL muscle, whole mount stained with NF/Syn (green) and α-BTX (red). Square, enlarged NMJs in right panel. Arrows, fragmented NMJs. Scale bars, 50 μm and 20 μm. **g-i** Quantification of data in (**f**). **g** More fragmentation in 12 M and 28 M-old mice; $t_{(18)} = 3.795$, **$p = 0.0013$; $t_{(18)} = 3.977$, ***$p = 0.0009$. **h** Reduced endplate area per NMJ in aged mice; $t_{(18)} = 3.678$, **$p = 0.0017$; $t_{(18)} = 2.465$, *$p = 0.024$. **i** Reduced AChR cluster intensity in aged mice; $t_{(18)} = 4.74$, ***$p = 0.0002$; $t_{(18)} = 4.198$, ***$p = 0.0005$. n = 10 NMJs from 3 mice per group, unpaired two-tailed *t* test in (**g-i**). Data

are shown as mean ± SEM. Source data are provided as a Source Data file.

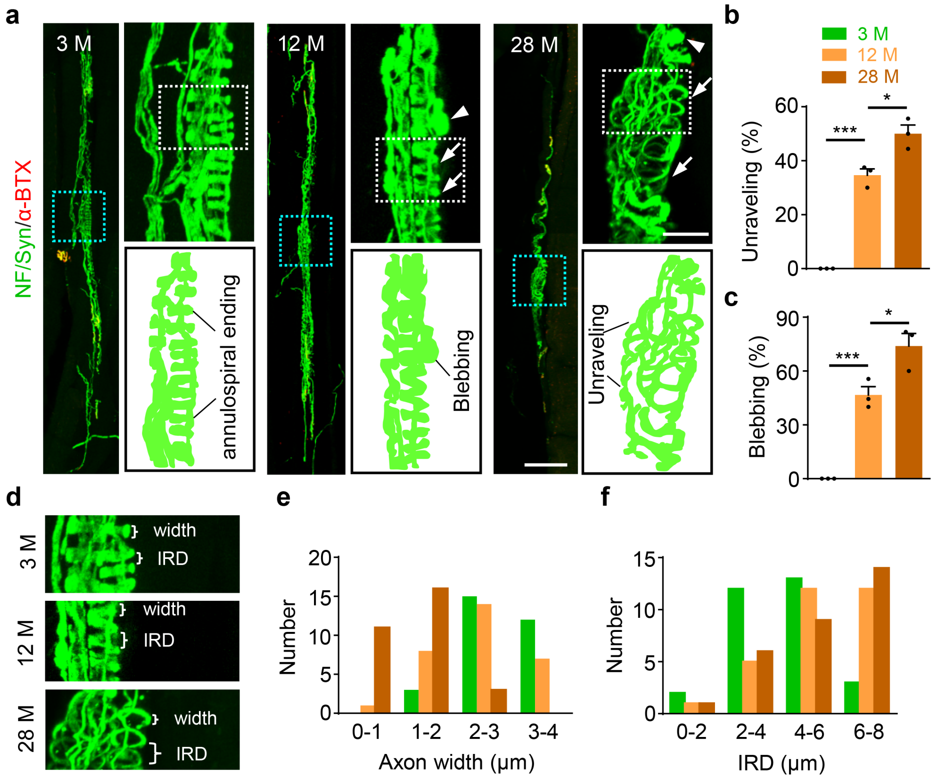

Supplementary Fig. 6 Deformation of muscle spindles in the aged mice. a Deformation of muscle spindle in the aged mice. Representative images of muscle spindle in EDL, whole mount stained with NF/Syn (green) and α-BTX (red) at 3 M, 12 M and 28 M-old. Bulbs (arrowheads) were observed and the annulospiral endings structure was altered (arrows) in aged muscle spindle. Light blue squares, enlarged views on the side. White squares, images shown in (**d**). Scale bars, 100 μm and 20 μm. **b, c, e, f** Quantification of data in (**a**). **b** Increased percentage of muscle spindle with unraveling in aged mice; $t_{(4)}$ = 14.811, ***p = 0.0001; $t_{(4)}$ = 3.851, *p = 0.0183. n = 3 mice per group, unpaired two-tailed t test. **c** Increased percentage of muscle spindle with blebbing in each group. Quantification of incidence of axon with large blebs in different age mice; $t_{(4)}$ = 10.088, ***p = 0.0005; $t_{(4)}$ = 3.254, *p = 0.0312. n = 3 mice per group, unpaired two-tailed t test. **d** Representative axon width and inter-rotational distance (IRD) in each group. **e** Thinner axons in aged mice. Quantification of mean axon width in muscle spindle in each group. **f** Larger IRD in aged mice. Quantification of IRD between annulospiral endings in muscle spindle in each group. Data are shown as mean ± SEM. Source data are provided as a Source Data file.

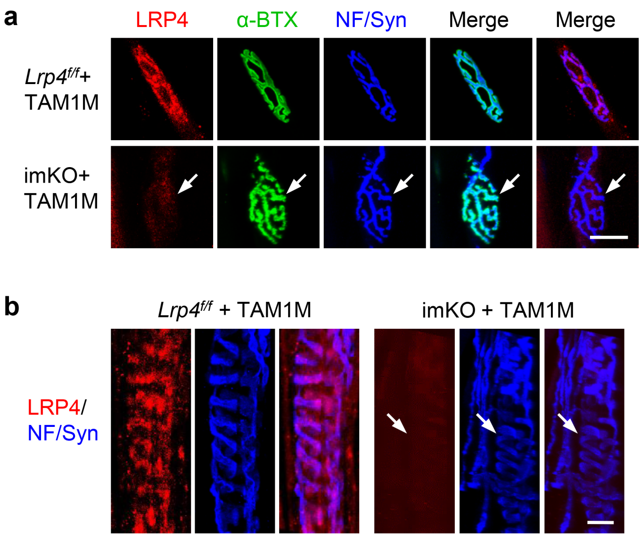

Supplementary Fig. 7 Specificity of 555-labeled LRP4 antibody in immunostaining. a, b EDL muscles whole mount stained with 555-labeled LRP4 (red), NF/Syn (blue) and CF488-labeled α-BTX (green). **a** LRP4 was located in NMJs in *Lrp4*$^{f/f}$ mice (P60), but absent from imKO mice treated with tamoxifen. Scale bar, 20 μm. **b** LRP4 was undetectable in muscle spindle in tamoxifen treated imKO mice. Arrows, LRP4 negative at NMJs and muscle spindle. Scale bar, 20 μm. These experiments (**a, b**) were repeated three times independently with similar results.
