# Supplementary material for: Intrafusal-fiber LRP4 for muscle spindle formation and maintenance in adult and aged animals
Source: Nat Commun. 2023 Feb 10;14:744. doi: 10.1038/s41467-023-36454-8 (PMC9918736; doi:10.1038/s41467-023-36454-8)
Supplement: Supplementary file 3 — Reporting Summary [file 41467_2023_36454_MOESM3_ESM.pdf]

nature portfolio

Corresponding author(s): Wen-Cheng Xiong and Lin Mei

Last updated by author(s): Jan 16, 2023

Reporting Summary

Nature Portfolio wishes to improve the reproducibility of the work that we publish. This form provides structure for consistency and transparency in reporting. For further information on Nature Portfolio policies, see our Editorial Policies and the Editorial Policy Checklist.

Please do not complete any field with "not applicable" or n/a. Refer to the help text for what text to use if an item is not relevant to your study.
For final submission: please carefully check your responses for accuracy; you will not be able to make changes later.

Statistics

For all statistical analyses, confirm that the following items are present in the figure legend, table legend, main text, or Methods section.

n/a	Confirmed	
☐	☒	The exact sample size (*n*) for each experimental group/condition, given as a discrete number and unit of measurement
☐	☒	A statement on whether measurements were taken from distinct samples or whether the same sample was measured repeatedly
☐	☒	The statistical test(s) used AND whether they are one- or two-sided<br>*Only common tests should be described solely by name; describe more complex techniques in the Methods section.*
☐	☒	A description of all covariates tested
☐	☒	A description of any assumptions or corrections, such as tests of normality and adjustment for multiple comparisons
☐	☒	A full description of the statistical parameters including central tendency (e.g. means) or other basic estimates (e.g. regression coefficient) AND variation (e.g. standard deviation) or associated estimates of uncertainty (e.g. confidence intervals)
☐	☒	For null hypothesis testing, the test statistic (e.g. *F*, *t*, *r*) with confidence intervals, effect sizes, degrees of freedom and *P* value noted<br>*Give P values as exact values whenever suitable.*
☒	☐	For Bayesian analysis, information on the choice of priors and Markov chain Monte Carlo settings
☒	☐	For hierarchical and complex designs, identification of the appropriate level for tests and full reporting of outcomes
☒	☐	Estimates of effect sizes (e.g. Cohen's *d*, Pearson's *r*), indicating how they were calculated

Our web collection on statistics for biologists contains articles on many of the points above.

Software and code

Policy information about availability of computer code

Data collection	Data and images were collected using Zen 2.3 system (Zeiss LSM 810) and ImageJ v1.52 (National Institute of Health).
Data analysis	The statistical analysis was performed using Graphpad Prism 6.0 (Graphpad Software).

For manuscripts utilizing custom algorithms or software that are central to the research but not yet described in published literature, software must be made available to editors and reviewers. We strongly encourage code deposition in a community repository (e.g. GitHub). See the Nature Portfolio guidelines for submitting code & software for further information.

Data

Policy information about availability of data

All manuscripts must include a data availability statement. This statement should provide the following information, where applicable:

- Accession codes, unique identifiers, or web links for publicly available datasets
- A description of any restrictions on data availability
- For clinical datasets or third party data, please ensure that the statement adheres to our policy

All data supporting the findings of this study are available in the article and its supplementary information. Source data are provided with this paper. The source data underlying Fig.1j, 2c, d, f, g, 3d-g, 4c-g, 5c, e, f, h, j, k, 6c, e, g-k, 7g-j, 8b, c, e-g, i-l; Supplementary Fig. 1b, 2b-e, 3c-e, 4b-d, 5a-c, e, g-i, 6b, c, e, f, as well as uncropped gel or blots underlying Fig. 7a, b, 8d; Supplementary Fig. 2a, 4e are provided as Source Data file.

Human research participants

Policy information about studies involving human research participants and Sex and Gender in Research.

Reporting on sex and gender	Not applicable
Population characteristics	Not applicable
Recruitment	Not applicable
Ethics oversight	Not applicable

Note that full information on the approval of the study protocol must also be provided in the manuscript.

Field-specific reporting

Please select the one below that is the best fit for your research. If you are not sure, read the appropriate sections before making your selection.

☒ Life sciences ☐ Behavioural & social sciences ☐ Ecological, evolutionary & environmental sciences

For a reference copy of the document with all sections, see nature.com/documents/nr-reporting-summary-flat.pdf

Life sciences study design

All studies must disclose on these points even when the disclosure is negative.

Sample size	Sample sizes were determined based on previous similar experiments (Oliveira Fernandes M & Tourtellotte WG, J Neurosci 2015, 35, 5566; Wu HT et al., Neuron 2012, 75, 94) and by sample size calculator at ClinCalc.com (http://clincalc.com/Stats/SampleSize.aspx). The exact number of mice was indicated in the figure legends.
Data exclusions	No data were excluded from analysis.
Replication	To achieve meaningful statistical differences, 10 mice per group were used in behavior test (rotarod test and balance beam walk), as indicated in the figure legends. For isolated muscle staining ,tissue section staining or CTB injection experiments, at least 3 mice per group were used. For western blot or quantitative PCR, three independent replicates have been performed. All attempts at replication were successful.
Randomization	Mice used in this study were randomly assigned to each group to maintain randomization. For in vitro experiments, the samples were prepared and treated in random order.
Blinding	Investigators were blinded to groups allocation during data collection and data analysis.

Reporting for specific materials, systems and methods

We require information from authors about some types of materials, experimental systems and methods used in many studies. Here, indicate whether each material, system or method listed is relevant to your study. If you are not sure if a list item applies to your research, read the appropriate section before selecting a response.

Materials & experimental systems

n/a	Involved in the study	
☐	☒	Antibodies
☐	☒	Eukaryotic cell lines
☒	☐	Palaeontology and archaeology
☐	☒	Animals and other organisms
☒	☐	Clinical data
☒	☐	Dual use research of concern

Methods

n/a	Involved in the study	
☒	☐	ChIP-seq
☒	☐	Flow cytometry
☒	☐	MRI-based neuroimaging

Antibodies

Antibodies used	The information of primary antibodies used was as follows: CF568-labeled α-bungarotoxin (α-BTX, Biotium Cat# 0006, 1:1000 for staining); CF488A-labeled α-bungarotoxin (488-α-BTX, Biotium Cat# 0005, 1:1000 for staining); rabbit anti-neurofilament (NF, Cell Signaling Technology Cat# C28E10, 1:1000 for staining); rabbit anti-synapsin (Syn, Cell Signaling Technology Cat# D12G5, 1:1000 for

staining); mouse anti-myosin heavy chain (Developmental Studies Hybridoma Bank Cat#S46, 1:200 for staining); mouse anti-Egr3 (Santa Cruz Cat# sc-390967, 1:500-1000 for staining); rabbit anti-Parvalbumin (Swant Cat#PV25, 1:1000 for staining); guinea pig anti-VGluT1 (Sigma- Aldrich Cat# AB5905, 1:1000 for staining); goat anti-cholera toxin subunit B (CTB) (Sigma-Aldrich Cat#227040, 1:1000 for staining); mouse anti-LRP4 (UC Davis/ NIH NeuroMab Facility Cat# 75-221, 1:1000 for WB, 1:200 for staining); mouse anti-APP (6E10) (BioLegend Cat# 803001, 1:1000 for staining); rabbit anti-APP (Sigma-Aldrich Cat# A8717, 1:1000 for WB); rabbit anti-Flag (Sigma-Aldrich Cat# F7425, 1:2000 for WB); mouse anti-GAPDH (Novus Biologicals Cat# NB 600-502, 1:3000 for WB); mouse anti-β-actin (Cell Signaling Technology Cat# 3700, 1:5000 for WB). The information of secondary antibodies used were as follows: Alexa Fluor 488 donkey anti-rabbit IgG (Cat# 711-547-003); Alexa Fluor 647 donkey anti-guinea pig (Cat# 706-605-148); Alexa Fluor 647 donkey anti-rabbit (Cat# 711-605-152); Alexa Fluor 594 donkey anti-mouse (Cat# 715-585-150); Alexa Fluor 594 donkey anti-Goat (Cat# 705-585-003) were all diluted by 5% goat serum and 5% BSA in PBS (1:1000) and purchased from the Jackson ImmunoResearch. Horseradish peroxidase (HRP)-conjugated goat anti-rabbit IgG (Cat# 32260) and goat anti-mouse IgG antibodies (Cat# 32230) were from Thermo Fisher Scientific and used at 1:4000 for Western blot.

Validation

All antibodies have been validated by the manufacturers as shown on their websites.
CF568 or CF488A-labeled α-bungarotoxin were validated for mouse in immunofluorescence staining by references on the Biotium's website (https://biotium.com/product/a-bungarotoxin-cf-dye-other-conjugates/);
Rabbit anti-neurofilament was validated for mouse in immunofluorescence staining and immunohistochemistry staining by Cell Signaling Technology (https://www.cellsignal.cn/products/primary-antibodies/neurofilament-l-c28e10-rabbit-mab/2837);
rabbit anti-synapsin was validated for mouse in immunofluorescence staining, immunohistochemistry staining by Cell Signaling Technology (https://www.cellsignal.cn/products/primary-antibodies/synapsin-1-d12g5-xp-rabbit-mab/5297);
mouse anti-myosin heavy chain was validated for mouse in immunofluorescence and immunohistochemistry staining on the manufacturer's website (https://dshb.biology.uiowa.edu/S46);
mouse anti-Egr3 was validated for mouse in immunofluorescence and immunohistochemistry staining on the manufacturer's website (https://www.scbt.com/p/egr-3-antibody-a-7/);
rabbit anti-Parvalbumin was validated for mouse in immunohistochemistry staining by the manufacturer shown on the Labome's website (https://www.labome.com/product/SWant/PV-25.html);
guinea pig anti-VGluT1 was validated for mouse in immunohistochemistry staining on the manufacturer's website (https://www.merckmillipore.com/CN/zh/product/Anti-Vesicular-Glutamate-Transporter-1-Antibody,MM_NF-AB5905);
goat anti-cholera toxin subunit B was validated for mouse in immunohistochemistry staining by references on the manufacturer's website (https://www.sigmaaldrich.cn/CN/zh/product/mm/227040);
mouse anti-LRP4 was validated for mouse in western blot, immunocytochemistry, blocking or activating experiments by our earlier publication (Sun XD et al., Nat Neurosci 2016, 19: 1010-8; western blot knockout validation) and the manufacturer shown on the Labome's website (https://www.labome.com/product/Neuromab/75-221.html);
mouse anti-APP (6E10) was validated for mouse/human in immunohistochemistry staining, western blot by references on the BioLegend's website (https://www.biolegend.com/en-us/products/purified-anti-beta-amyloid-1-16-antibody-11228);
rabbit anti-APP was validated for mouse/ human in western blot, immunohistochemistry staining by references on the manufacturer's website (https://www.sigmaaldrich.cn/CN/zh/product/sigma/a8717);
rabbit anti-Flag was validated for all species in immunoprecipitation, western blot and immunofluorescence on the manufacturer's website (https://www.sigmaaldrich.cn/CN/zh/product/sigma/f7425);
mouse anti-GAPDH was validated for mouse/human in western blot on the manufacturer's website (https://www.novusbio.com/products/gapdh-antibody-6c5cc_nb600-502);
mouse anti-β-actin was validated for mouse/human in western blot on the manufacturer's website (https://www.cellsignal.com/products/primary-antibodies/b-actin-8h10d10-mouse-mab/3700);
the validation of fluoresce-conjugated secondary antibodies could be found on the website of Jackson ImmunoResearch (https://www.jacksonimmuno.com/);
the validation of HRP-conjugated secondary antibodies could be found on the website of Thermo Fisher Scientific (https://www.thermofisher.cn/antibody/secondary/query/HRP).

Eukaryotic cell lines

Policy information about cell lines and Sex and Gender in Research

Cell line source(s)	The embryo kidney cell line HEK293T was obtained from ATCC (Cat# CRL-3216).
Authentication	HEK293T has been authenticated by STR profiling by ATCC. The cell line was obtained from ATCC and examined of morphology and growth characteristics. The cell line was kept at low passages in the study in order to maintain their identity.
Mycoplasma contamination	HEK293T was tested to be mycoplasma negative.
Commonly misidentified lines (See ICLAC register)	No commonly misidentified cell lines were used in this study.

Animals and other research organisms

Policy information about studies involving animals; ARRIVE guidelines recommended for reporting animal research, and Sex and Gender in Research

Laboratory animals

Both sexes of adult Lrp4 CreERT2, Ai9, Lrp4 Lac Z, Lrp4f/f, HSA CreERT2, Flag-Lrp4 (3 M, 12 M, 24 M-old), APP null and C57BL/6 (3 M, 12 M, 24 M, 28 M-old) were used in this study. Older mice (12 M, 24 M, 28 M-old) were acquired from the National Institute on Ageing. Ai9 (Rosa-CAG-LSL-tdTomato) reporter mice (Stock No: 007905), APP null transgenic mice (B6.129S7-Apptm1Dbo/J, Stock No: 004133, C57 BL/6J background) were purchased from The Jackson Laboratory. HSA-Cre ERT2 mice were generated and described by Schuler M. et al., Genesis, 2005, 41, 165. Lrp4 CreERT2 was generated by lab and described in earlier paper (Zhang HS et al., J Neurosci 2020, 40, 5347). Lrp4 Lac Z reporter mice were from KOMP (VG15248) and described by Sun XD et al., Nat Neurosci 2016, 19, 1010. Lrp4f/f mice were generated by lab and described by Wu HT et al., Neuron 2012, 75, 94. Flag-Lrp4 mice were generated by

	lab and described by Zhao K et al., J Neurosci 2018, 38, 8860. Mice were housed no more than five per cage in a room with a 12 h light/dark cycle with ad libitum access to water and rodent chow diet (Diet 7097, Harlan Teklad). Mice were kept at an ambient temperature of 23 °C and humidity of 40-60%. The age of mice used in each experiments were described in figure legends. Experimenters were blinded to genotypes and treatments.
Wild animals	No wild animals were used in the study.
Reporting on sex	Used for experiments were male and female mice and embryos or neonatal mice of either sex.
Field-collected samples	No field collected samples were used in the study.
Ethics oversight	All animal experiments were approved by the Institutional Animal Care and Use Committee of Case Western Reserve University.

Note that full information on the approval of the study protocol must also be provided in the manuscript.

This checklist template is licensed under a Creative Commons Attribution 4.0 International License, which permits use, sharing, adaptation, distribution and reproduction in any medium or format, as long as you give appropriate credit to the original author(s) and the source, provide a link to the Creative Commons license, and indicate if changes were made. The images or other third party material in this article are included in the article's Creative Commons license, unless indicated otherwise in a credit line to the material. If material is not included in the article's Creative Commons license and your intended use is not permitted by statutory regulation or exceeds the permitted use, you will need to obtain permission directly from the copyright holder. To view a copy of this license, visit http://creativecommons.org/licenses/by/4.0/
